# Closed-loop Neurostimulation: The Clinical Experience

**DOI:** 10.1007/s13311-014-0280-3

**Published:** 2014-05-22

**Authors:** Felice T. Sun, Martha J. Morrell

**Affiliations:** 1 NeuroPace Inc., 445 N. Bernardo Avenue, Mountain View, CA 94043 USA; 2 Stanford University, Neurology, Stanford, CA USA

**Keywords:** Responsive, adaptive, stimulation, pain, epilepsy, Parkinson disease

## Abstract

Neurostimulation is now an established therapy for the treatment of movement disorders, pain, and epilepsy. While most neurostimulation systems available today provide stimulation in an open-loop manner (i.e., therapy is delivered according to preprogrammed settings and is unaffected by changes in the patient’s clinical symptoms or in the underlying disease), closed-loop neurostimulation systems, which modulate or adapt therapy in response to physiological changes, may provide more effective and efficient therapy. At present, few such systems exist owing to the complexities of designing and implementing implantable closed-loop systems. This review focuses on the clinical experience of four implantable closed-loop neurostimulation systems: positional-adaptive spinal cord stimulation for treatment of pain, responsive cortical stimulation for treatment of epilepsy, closed-loop vagus nerve stimulation for treatment of epilepsy, and concurrent sensing and stimulation for treatment of Parkinson disease. The history that led to the development of the closed-loop systems, the sensing, detection, and stimulation technology that closes the loop, and the clinical experiences are presented.

**Electronic supplementary material:**

The online version of this article (doi:10.1007/s13311-014-0280-3) contains supplementary material, which is available to authorized users.

## Introduction

In the last 2 decades, since the US Food and Drug Administration (FDA) approval of deep brain stimulation (DBS) for the treatment of tremor in 1997 [1], neurostimulation has become an important therapeutic option for patients with diseases of the nervous system. Neurostimulation is now an established therapy for treatment of essential tremor [2], Parkinson disease (PD) [3, 4], epilepsy [5, 6], and neuropathic pain [7, 8], and is being investigated for numerous other neurologic and psychiatric disorders, including memory disorders, depression, obsessive–compulsive disorder, and Tourette syndrome [9, 10].

Most neurostimulation systems available today provide stimulation in an open-loop manner, which means that stimulation settings are preprogrammed and do not automatically respond to changes in the patient’s clinical symptoms or in the underlying disease. While open-loop stimulation paradigms are effective, limitations of open-loop stimulation have become more evident as clinical experience grows. For example, although open-loop spinal cord stimulation systems are generally effective for the treatment of pain, open-loop systems may provide too much or too little therapy because the stimulation settings are not automatically adjusted based on the patient’s body position [11–13]. A closed-loop system may provide improved and more consistent pain relief by automatically adjusting the stimulation settings according to the patient’s body position. In another example, open-loop DBS systems for the treatment of PD, while effective in managing the motor symptoms, may be inefficient because the same level of stimulation is provided regardless of the extent of motor impairment [14]. A closed-loop system may provide stimulation more efficiently by delivering stimulation only when motor function is impaired.

Advancements in implantable technology have led to the design and clinical introduction of implantable closed-loop neurostimulation systems that continuously sense physiological signals, detect prespecified physiological changes, and adjust therapy in response to the detected signals. Closed-loop therapies may offer advantages relative to open-loop therapies by increasing the efficacy of stimulation [14–16], improving the clinical benefit of stimulation [16], and reducing the side effects of stimulation [14].

This review focuses on the clinical experience of implantable closed-loop neurostimulation systems for treatment of pain, epilepsy, and movement disorders. The history that led to the development of the closed-loop system, the sensing, detection, and stimulation technology that closes the loop, and the clinical experience are presented.

## Pain

### Background

Neurostimulation to treat pain was introduced clinically in 1967, when the first spinal cord stimulator was implanted to stimulate the dorsal columns of the spinal cord [17]. Since then, numerous technological developments, including improved lead design and advanced programming capabilities, have improved the efficacy of the therapy. Spinal cord stimulation (SCS) is now a widely accepted form of therapy for chronic intractable neuropathic pain and is the most commonly employed neurostimulation therapy for treatment of pain [18].

SCS provides pain relief by interfering with pain signals traveling along the spinal cord. To achieve optimum efficacy, the stimulation settings must be adjusted for each patient. Stimulation amplitude that is too low may be ineffective, and stimulation amplitude that is too high may be perceived as painful [11].

One of the challenges of SCS systems is to provide effective pain relief in all body positions. Because the stimulating electrodes are typically situated in the epidural space, a change in body position may change the epidural distance or the longitudinal distance, which may result in a change in the location and intensity of the stimulation-induced effect [11–13, 19]. For example, the same stimulation settings that are effective when the patient is standing may be too intense when the patient is lying down.

**Fig. 1:**
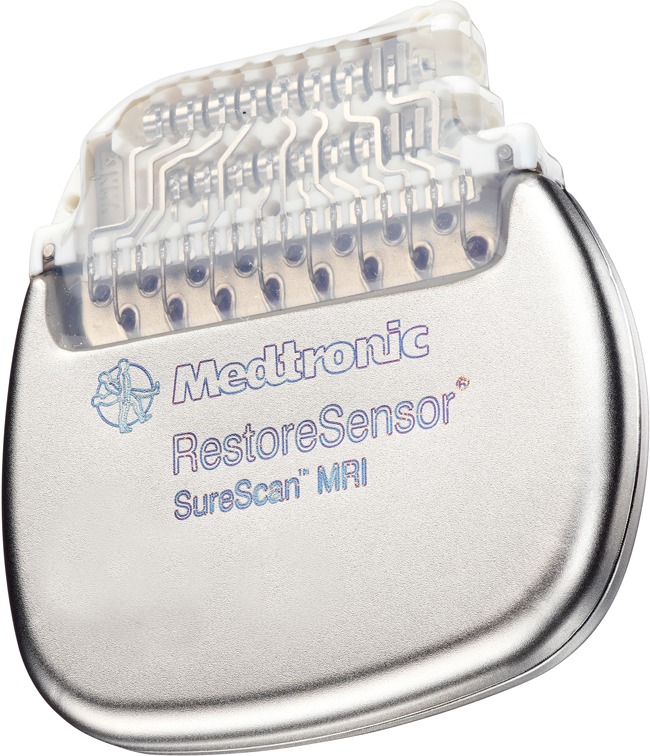
The Medtronic RestoreSensor SureScan MRI SCS device (Medtronic, Minneapolis, MN, USA). Reprinted with the permission of Medtronic, Inc. © 2013

Open-loop SCS systems address this issue using a patient-controlled programmer that allows the patient to adjust the stimulation intensity within clinician-prescribed limits. However, the patient may need to make multiple adjustments during the day to maintain adequate pain control. Recently, a closed-loop SCS system, the RestoreSensor system (Medtronic, Minneapolis, MN, USA), has been approved by the US FDA to provide automatic adjustments in stimulation according to the patient’s body position (Fig. 1).

### Closed-loop Technology

The RestoreSensor system uses a 3-axis accelerometer that senses the patient’s body position and activity, and then adjusts the stimulation settings according to the body position. In order to provide this closed-loop therapy, the RestoreSensor neurostimulator must first be trained to recognize different body positions. During this process, the patient is positioned in multiple orientations such as upright, lying prone, lying supine, lying on the left side, and lying on the right side. Data from the 3-axis accelerometer are linked to each of these positions, and patient-specific stimulation settings are optimized for each position.

Once the position-adaptive stimulation feature is programmed, the system detects changes in body position and activity in real time and automatically adjusts stimulation according to the preprogrammed settings.

### Clinical Experience

Two clinical studies assessed safety and efficacy of closed-loop SCS for pain [16, 20].

The first evaluated the feasibility and utility of an accelerometer-based algorithm to automatically adjust stimulation settings based on body position or activity [20]. This prospective, open-label, randomized study enrolled 20 patients across 2 centers. Fifteen participants completed the in-clinic protocol to provide data for the study.

Prior to entering this study, patients had already been implanted with either the Restore or RestoreAdvanced spinal cord stimulator (Medtronic) for at least 3 months, demonstrated stable pain control with their neurostimulation system, and used their patient programmer to change the stimulation amplitude in response to body position or activity at least twice a day during a 3-day baseline period.

During the in-clinic phase, the patient’s baseline stimulation settings were recorded. Additionally, stimulation thresholds were obtained for 8 different positions: standing, sitting, lying supine, lying on right side, lying prone, lying on left side, reclining at 45°, and walking on a treadmill. An external sensor containing a triaxial accelerometer was then fitted to the patient, and accelerometer measurements were taken for a subset of those positions. The external sensor communicated directly with a programmer to adjust the stimulation settings according to the measured body position.

Each patient self-rated their overall satisfaction for each of the 8 positions when stimulation was adjusted manually and with two different automatic stimulation adjustment modes. Patients reported significantly higher satisfaction using the automatic stimulation adjustment modes compared with the manual adjustment mode, and 74 % of patients reported that stimulation settings using the automatic adjustment algorithm were “just right” (*vs* “too high” or “too low”). This study demonstrated the feasibility and utility of using a triaxial accelerometer to measure the body position in order to automatically adjust the stimulation settings.

The automatic adjustment algorithm was subsequently evaluated in a clinical study of an implantable spinal cord neurostimulation system [16]. A prospective, multicenter, open-label, randomized crossover study was conducted in 79 patients implanted with the RestoreSensor (Medtronic) SCS system to assess whether the position-adaptive stimulation feature provided benefit in terms of pain relief and/or convenience compared with not using the feature.

For the first 4 weeks after implantation of the system, only manual adjustment of stimulation was enabled. Patients were then randomized 1:1 to receive either position-adaptive stimulation or conventional manual programming for 6 weeks, and were then crossed over to receive the other type of stimulation for 6 weeks.

In the intent-to-treat analysis, 86.5 % of patients achieved the primary objective of improved pain relief with no loss of convenience, or had improved convenience with no loss of pain relief using automatic position-adaptive stimulation compared with using conventional manual programming adjustment alone. Moreover, patients reported improved comfort during position changes (80.3 %), improved activity (69.0 %), and improved sleep (47.9 %) with position-adaptive stimulation. There were 25 adverse events (AEs) related to undesirable changes in stimulation; however, only 9 of the events occurred during the position-adaptive stimulation arm of the study [16].

These studies illustrate how a fairly straightforward and simple closed-loop system using body orientation as a feedback signal significantly improved the patient experience. Closed-loop position-adaptive stimulation improved patient-reported pain relief, activity, sleep, and convenience compared with using manual programming adjustment alone. Future SCS systems may employ more sophisticated closed-loop algorithms, such as using electrically evoked compound action potentials to automatically adjust stimulation parameters, which could allow for even more effective and efficient pain control [21].

## Epilepsy

The earliest report of applying electrical stimulation to the brain to treat seizures in humans is by Penfield and Jasper in 1954 [22]. In their acute experiments, they observed that in some cases electrical stimulation of the cortex resulted in a flattening of the local electrocorticogram (both normal rhythms and spontaneous epileptiform discharges).

Numerous studies published over the last 6 decades have evaluated the safety and efficacy of brain stimulation to treat epilepsy [23]. Promising results were reported for stimulation of the cerebellum [24–26], caudate nucleus [27–29], centromedian nucleus [28, 30, 31], subthalamic nucleus (STN) [32], hippocampus [33–35], and the anterior nucleus of the thalamus [36–38]. However, with the exception of a randomized double-blinded controlled trial of stimulation of the anterior nucleus of the thalamus as adjunctive treatment of medically intractable partial onset seizures in adults [38], these studies were largely uncontrolled, small, and have not been replicated.

There are currently 2 neurostimulation therapies approved by the US FDA for treatment of epilepsy: open-loop vagus nerve stimulation (VNS Therapy Cyberonics, Houston, TX, USA) and closed-loop responsive cortical stimulation (RNS System, NeuroPace, Mountain View, CA, USA).

The following two sections describe the background, the closed-loop technology, and clinical experience of the US FDA-approved closed-loop responsive cortical stimulation system and an investigational closed-loop vagus nerve stimulation system, which builds on the approved open-loop vagus nerve stimulation technology.

### Closed-loop Responsive Cortical Stimulation

#### Background

Initial experiments assessed the safety and efficacy of responsive electrical stimulation for epilepsy during in-patient intracranial monitoring to determine whether the patient was a candidate for epilepsy surgery. A routine procedure during this evaluation is to deliver focal cortical stimulation to identify functional and epileptogenic regions. At times, the electrical stimulation elicits afterdischarges (ADs), which are rhythmic discharges that are similar to spontaneous epileptiform activity. Lesser et al. [39] observed that a brief burst of electrical stimulation (similar to the stimulation used to elicit ADs) could also terminate an AD. The most effective stimulation to terminate ADs was brief (0.5–1 s), applied early (within 4.5 s of the AD), and applied at the site of the AD [39, 40].

This work paved the way for the development of closed-loop responsive stimulation systems designed to deliver electrical stimulation in response to spontaneous epileptiform activity. The first such systems were large, nonimplantable systems, developed as proof-of-concept prototypes to assess whether closed-loop responsive stimulation was feasible and could be effective [41, 42].

Peters et al. [41] used an external bedside system that performed real-time seizure detection and automatically delivered electrical stimulation in response to the seizure detection. This system was evaluated in 8 patients undergoing intracranial monitoring [43]. Four patients received responsive stimulation directly to the epileptogenic zone (local closed-loop) and 4 received responsive stimulation to the anterior thalami (remote closed-loop). The mean reduction in seizures in the local closed-loop stimulation group was 55.5 % and 40.8 % in the remote closed-loop group. Four of the 8 patients had a ≥ 50 % reduction in seizures with responsive stimulation (3 with local closed-loop stimulation and 1 with remote closed-loop stimulation). Kossoff et al. [42] also reported on the experience using an external responsive neurostimulator in 4 patients. Stimulation delivered in response to epileptiform activity appeared to reduce the number of clinical seizures and to suppress electrographic seizures. These studies demonstrated that closed-loop responsive stimulation was feasible and provided preliminary evidence that responsive stimulation could reduce seizures.

#### Closed-loop Technology

**Fig. 2:**
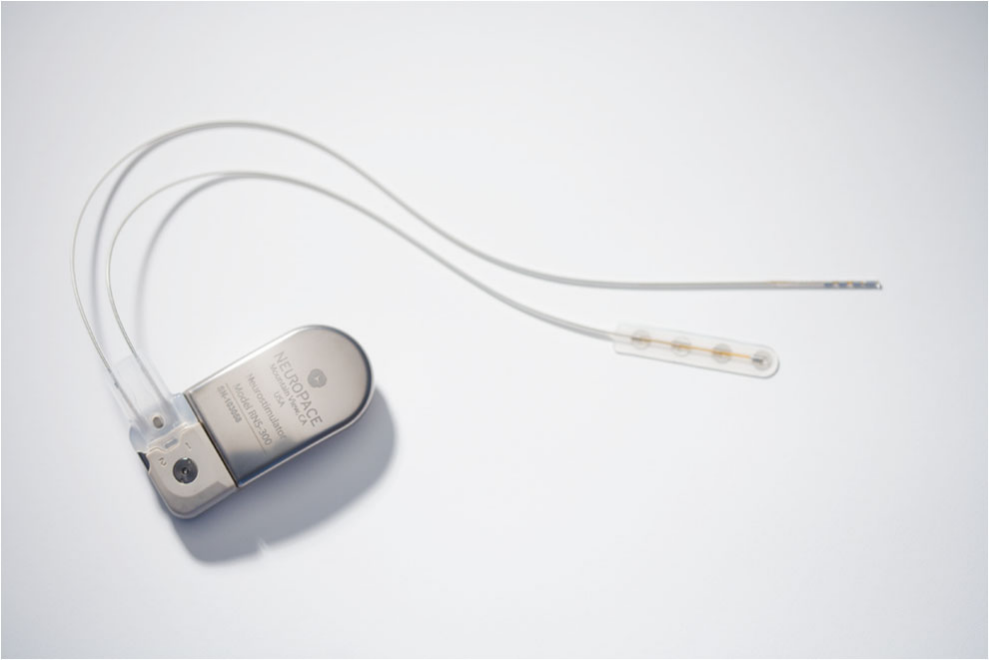
The RNS Neurostimulator connected to the NeuroPace Depth Lead and NeuroPace Cortical Strip Lead (NeuroPace, Mountain View, CA, USA)

The RNS System is a responsive cortical neurostimulator system approved by the US FDA as an adjunctive therapy in reducing the frequency of seizures in individuals aged 18 years or older with partial onset seizures who have undergone diagnostic testing that localized no more than 2 epileptogenic foci, are refractory to ≥ 2 antiepileptic medications, and currently have frequent and disabling seizures (motor partial seizures, complex partial seizures, and/or secondarily generalized seizures). The RNS System includes a neurostimulator that is implanted in the cranium and connected to 1 or 2 recording and stimulating depth and/or cortical strip leads that are surgically placed in the brain at the seizure foci (Fig. 2). The closed-loop neurostimulator provides responsive electrical stimulation directly to 1 or 2 seizure foci when abnormal electrocorticographic activity is detected. The system includes a programmer for the physician, a remote monitor for the patient, and a secure internet-accessed database for storage of neurostimulator data obtained by the programmer or remote monitor.

### Sensing and Data Storage

The neurostimulator continuously senses and monitors electrographic activity through the implanted cortical depth and strip leads. The neurostimulator records the date and time of all detections and stimulations, and stores segments of the electrographic data for review by the physician. Electrographic data storage is triggered by detection, responsive stimulation, scheduled time of the day, magnet (used by the patient to indicate a seizure), and/or other events as programmed by the physician. These data allow physicians to assess detection sensitivity and effects of stimulation.

### Detection

The detection algorithms in the neurostimulator are computationally efficient and optimized in order to perform real-time detection within the constraints of currently available implantable technology, such as limited power and processing capabilities [44]. Three detection tools (bandpass, line-length, and area) are provided. The detection parameters are highly configurable and are selected by the physician to adjust the sensitivity, specificity, and latency of the detection.

The bandpass tool is similar to that described by Gotman [45], and is used to detect spikes and rhythmic activity occurring in specific frequency ranges. This tool uses “half-waves”, which are segments of the electrographic signal partitioned at local minima and maxima. The amplitude and duration of the half-waves are representative of the amplitude and frequency components of the electrographic signal.

The line-length algorithm, described by D’Alessandro et al. [46] and Esteller et al. [47] identifies changes in both amplitude and frequency. The line-length is defined as the average of absolute sample-to-sample amplitude differences within a window. A short-term sliding window average (128 ms–4 s) is compared with a long-term sliding window average (4 s–16 min). Detection occurs when the short-term measurement crosses an absolute or relative threshold derived from the long-term measurement.

The area feature is similar to an energy or power measurement and identifies changes in overall signal energy without regard for frequency [48–50]. Area is defined as the average absolute area-under-the-curve of the electrographic signal. As with line-length, a short-term window average is compared with a long-term background window average, and detection occurs when a previously defined threshold is crossed.

These 3 detection algorithms are efficient (requiring low computational power), and can be configured to detect electrographic events within a fraction of a second or to detect more subtle changes in amplitude, frequency, and/or power that occur over several seconds.

### Stimulation

The neurostimulator delivers current-controlled, charge-balanced biphasic pulses, and is programmed by the physician to deliver stimulation frequencies ranging from 1 to 333 Hz, current amplitudes from 0.5 to 12.0 mA, and pulse-widths from 40 to 1000 μs. The stimulation montage can be configured to deliver current between any combination of electrodes, including the neurostimulator case.

### Clinical Use

The RNS System is not a seizure predictor and cannot determine if the patient has a clinical seizure. In the clinical experience to date, the neurostimulator has typically been programmed to detect spike and slow waves, rhythmic changes in frequency, or changes in amplitude typical of the electrocorticographic features that sometimes progressed to an electrographic and/or clinical seizure. The most common stimulation programming was an amplitude of 1.5–3.0 mA, a pulse width of 160 μs, a stimulation burst duration of 100–200 ms, and a pulse frequency of between 100 and 200 Hz. The majority of patients received stimulation in response to detections 600–2000 times a day for a cumulative total of < 5 mins of stimulation over 24 h. As this closed-loop stimulation was in response to detections of specific electrocorticographic patterns, and it was not possible to know which of these detections would have progressed to an electrographic (or clinical) seizure, the concept of false-positive or false-negative seizure detections was therefore not relevant to this stimulation approach.

#### Clinical Experience

Safety and efficacy of the RNS System as an adjunctive treatment in adults with medically intractable partial-onset seizures was established in 3 clinical trials: a 2-year primarily open-label safety study (feasibility study, *n* = 65), a 2-year double-blinded randomized sham-stimulation controlled study (pivotal study, *n* = 191), and a long-term extension study (long-term treatment study) designed to collect an additional 7 years of efficacy and safety data in patients completing the feasibility or pivotal studies.

The pivotal study was a multicenter, double-blinded, randomized sham-stimulation controlled study to demonstrate the safety and effectiveness of the RNS System as an adjunctive treatment for adults with medically intractable partial-onset seizures arising from 1 or 2 seizure foci. Patients were, on average, 34.9 years old and had epilepsy for 20.5 years. Nearly one-third (32 %) had prior therapeutic epilepsy surgery (resection, subpial transection, and/or callosotomy), and more than a third (34 %) had previously been treated with a vagus nerve stimulator.

The effectiveness of responsive stimulation was assessed by comparing the seizure reduction in the group receiving active stimulation (treatment group) *versus* the group receiving no stimulation (sham group) during a 12-week blinded period relative to a preimplant baseline. The primary effectiveness endpoint was met: the reduction in seizure frequency in the treatment group (–37.9 %) was significantly greater than that in the Sham group (–17.3 %; *p* = 0.012) [6]. There was no difference in effectiveness in patients with mesial temporal lobe seizure onsets compared with patients with neocortical seizure onsets, in patients whose seizures arose from 1 compared with 2 foci, in patients who had been treated with vagus nerve stimulation (VNS) compared with those who had not, and in patients who had already undergone a therapeutic epilepsy surgery compared with those who had not.

During the open-label period of the study, when all patients had the opportunity to receive responsive stimulation, seizure reduction continued to improve. The median percent reduction in seizures was 44 % at 1 year and 53 % at 2 years postimplant compared with baseline [51].

Safety was assessed using AE data. The RNS System serious AE rate was no worse than the literature-derived serious AE rate for comparable procedures. Stimulation was well tolerated. There was no difference in the frequency or type of AE between the treatment and sham groups, except for side effects of antiepileptic medications, which were more common in the sham group (5 patients, all mild events) than the treatment group (none). (Refer to the RNS System product labeling for detailed disclosure of specific indications, contraindications, warnings, precautions, and AEs.)

Additional assessments included a quality of life inventory, neuropsychological evaluations, and mood inventories. At 1 and 2 years after implantation, patients reported significant improvements in overall quality of life (*p* < 0.001) and in 9 of the primary scale scores, including memory, concentration, and language [51]. There was no significant deterioration in any neuropsychological measures or any of the mood inventories at the end of the blinded period compared with the baseline period, or at 1 and 2 years postimplant, indicating there were no acute, delayed, or longer-term adverse effects of responsive stimulation on neuropsychological function and mood.

These results demonstrate that closed-loop responsive stimulation to the seizure focus can reduce the frequency of partial onset seizures, is well-tolerated, and is acceptably safe.

### Closed-loop VNS

#### Background

Open-loop VNS is approved by the US FDA for use as an adjunctive therapy in reducing seizure frequency in adults and adolescents over the age of 12 years with partial-onset seizures that are refractory to antiepileptic drugs. Intermittent scheduled stimulation is delivered to the vagus nerve. A typical stimulation schedule is 30 s “on” and 5 min “off,” but this can be adjusted by the physician. By moving a magnet over the VNS pulse generator, patients may also initiate on-demand stimulation bursts to provide additional VNS therapy during an aura or at the onset of a seizure.

Two randomized, blinded, active-control (high-stimulation/low-stimulation) trials evaluated the safety and efficacy of open-loop VNS therapy [5, 52]. The median percent reduction in daily seizures was 23–24 % in the high stimulation group and 6–21 % in the low stimulation group. The reduction in seizures in an open-label extension study was 31 % at 1 year and 41 % at 2 years. (Refer to the VNS therapy product labeling for detailed disclosure of specific indications, contraindications, warnings, precautions, and AEs.)

The concept of closed-loop VNS therapy emerged from the experience with magnet-activated stimulation, where a magnet is used by the patient or caregiver to trigger additional bursts of stimulation at the time of an aura or seizure onset. One prospective and 1 retrospective study evaluated magnet-activated VNS therapy [53, 54]. The first was a single-center, prospective study of 35 patients that was designed to assess the efficacy of magnet-activated stimulation [53]. After implantation of the VNS system, patients and their caregivers were provided with a magnet and instructed how to use the magnet to provide additional stimulation when an aura or seizure onset occurred. Of the 35 implanted patients, 21 (or their caregivers) were able to use the magnet and provide reliable seizure information. Fourteen of the 35 patients were unable to use the magnet: 9 because they had no auras or their seizures were too brief; 3 had become seizure-free before the magnet was provided; and 2 had unreliable data. Of the 21 patients who were able to use the magnet, 14 reported a positive effect of the magnet and 7 reported no effect of the magnet. Of note, only 3 patients were able to use the magnet themselves. In most cases, support from caregivers was necessary. Data from this study suggest that acute VNS delivered at the onset of a seizure may provide added benefit to the standard scheduled VNS, but reliable manual delivery of stimulation may be difficult to achieve.

A second, retrospective study evaluated magnet usage during two open-loop VNS trials: the E03 and E04 trials [54]. In the randomized, double-blind, controlled, E03 trial, patients were randomized 1:1 to receive therapeutic VNS therapy (treatment group) or nontherapeutic stimulation (active control group). During the blinded period of the trial, magnet-activated stimulation was on for the treatment group and off for the active control group. Of the 114 participants in the E03 study, 92 (or their caregivers) used the magnet (50 in the treatment group and 42 in the active control group). The treatment group reported that 21.3 % of the seizures that received magnet-activated stimulation were terminated compared with 11.9 % in the active-control group (*p* = 0.08). Additionally, the treatment group was more likely to report improvement with magnet usage compared with the active control group (*p* = 0.05).

During the open-label E04 trial, 86/124 participants used their magnets. Of the 86 patients (or caregivers) who used the magnets, 22 % reported seizure termination, 31 % reported seizure diminution, and 47 % reported no effect of magnet-activated VNS therapy. Patient-reported outcomes were available for 9482 seizures for which magnet-activated VNS was used: for 2211 seizures (24 %) magnet-activated VNS terminated the event, for 3638 seizures (38 %) magnet-activated VNS diminished the event, and for 3633 seizures (38 %) magnet-activated VNS did not affect the event.

Results from both studies of magnet-activated stimulation suggest that there could be a positive effect of additional VNS therapy during an aura or at the onset of a seizure. However, both studies also revealed that most patients were unlikely to be able to use the magnet on their own—a caregiver was typically involved in activating the additional stimulation. This led to the development of a closed-loop VNS system, the AspireSR (Cyberonics), which is an investigational vagus nerve stimulator that automates the delivery of additional stimulation using a cardiac-based seizure detector.

#### Closed-loop Technology

Cardiac-based seizure detection relies on changes in the heart rate at the onset or during a seizure. The most frequently reported type of seizure-related cardiac change is ictal tachycardia, or an increase in heart rate with seizure, which occurs in > 70 % of seizures [55–58]. Thus, several groups have proposed cardiac-based seizure detection based on this increase in heart rate [59–62].

Although the cardiac-based seizure detection algorithm for the AspireSR generator has not been published, one computationally efficient approach that could be performed in real-time in an implantable system is to track the heart rate using both long- and short-term trends [59]. The long-term heart rate trend represents the background rate, which may change slowly over time based on the patient’s activity level. The short-term heart rate trend represents the foreground rate. When the foreground heart rate exceeds a threshold relative to the background heart rate, an event is detected. Because the threshold is based on the background heart rate, it automatically adjusts to the patient’s underlying activity.

#### Clinical Experience

The AspireSR is a vagus nerve stimulator with a cardiac-based seizure detection feature. The performance of the cardiac-based seizure detection feature was evaluated in a study of 31 patients. Patients implanted with the AspireSR generator were observed in epilepsy monitoring units for up to 5 days to identify seizures and collect heart rate data. More than 80 % of seizures that were accompanied by ictal tachycardia were detected by the AspireSR generator. Thus, the primary endpoint of the study was met. The potential false detection rates were low. The detections occurred close, and in some cases prior, to seizure onset. This study demonstrated that the cardiac-based seizure detection algorithm implemented in the AspireSR system could detect seizures with heart rate changes [63].

### Movement Disorders

#### Background

DBS has been a treatment option for movement disorders since 1997 when the first DBS system was approved for treatment of tremor [1]. Over the last 2 decades, open-loop DBS has proven to be a valuable and effective therapy option for patients with PD, essential tremor, or dystonia whose motor symptoms cannot be controlled by drug therapy alone [3, 4, 64–67].

Despite the success of open-loop DBS for movement disorders, many believe that closed-loop technology can improve the therapy by potentially reducing the side effects of stimulation and/or improving the efficacy of stimulation. For example, side effects associated with DBS such as impairment of speech, gait, and balance may be ameliorated by a closed-loop responsive stimulation approach where stimulation is delivered intermittently [14, 68, 69]. Additionally, closed-loop responsive or adaptive stimulation may improve the efficacy of DBS [14, 15]. Indeed, Rosin et al. [15] discovered that delivering closed-loop stimulation to the internal segment of the globus pallidus in response to action potentials recorded in the primary motor cortex is more efficient and effective in alleviating Parkinsonian motor symptoms than continuous stimulation.

There are also other potential advantages of closed-loop stimulation. Closed-loop responsive stimulation may increase the battery longevity, thus exposing the patient to fewer neurostimulator replacement procedures. Additionally, closed-loop systems may facilitate the process of identifying the optimal stimulation settings by using a physiological signal as a biomarker to titrate the stimulation parameters, rather than relying on clinically overt symptoms [70, 71]. Thus, there is significant interest in developing closed-loop DBS systems for treatment of movement disorders.

A number of approaches have been proposed using various physiological signals for feedback in the closed-loop system, including electromyography [72–74] and single- or multi-unit recordings [15]. However, these signals are not amenable to chronic recording. Perhaps the most practical approach is to leverage existing open-loop neurostimulation systems and use local field potentials (LFPs) sensed from the stimulating electrode to close the loop [14, 75].

For treatment of PD, several groups have proposed using the power in the beta frequency band (13–30 Hz) of the LFP in the STN as a biomarker for closed-loop stimulation [76–78]. In patients with PD, there is often a prominence in the beta frequency band that is correlated with the severity of motor symptoms and is suppressed with levodopa treatment [79], as well as with DBS [76, 79, 80]. Moreover, the suppression of the beta activity is correlated with improved motor performance [76]. Therefore, the beta band may be a useful biomarker for the severity of motor symptoms and the efficacy of stimulation.

Little et al. [14] provided a proof-of-principle of this approach. In 8 patients undergoing DBS implantation for PD, the LFP recorded from the stimulating electrode implanted in the STN was used to trigger closed-loop stimulation. Specifically, the LFP was filtered and rectified to produce an “online” value of beta amplitude. This signal was used to trigger stimulation via a user-defined threshold such that stimulation was delivered approximately 50 % of the time. Stimulation, once triggered, was sustained until the beta amplitude fell below the threshold. The authors reported that this closed-loop stimulation resulted in improved motor response relative to continuous open-loop stimulation. This research demonstrates that the LFP in the STN could be a useful biomarker to control DBS.

#### Closed-loop Technology

**Fig. 3:**
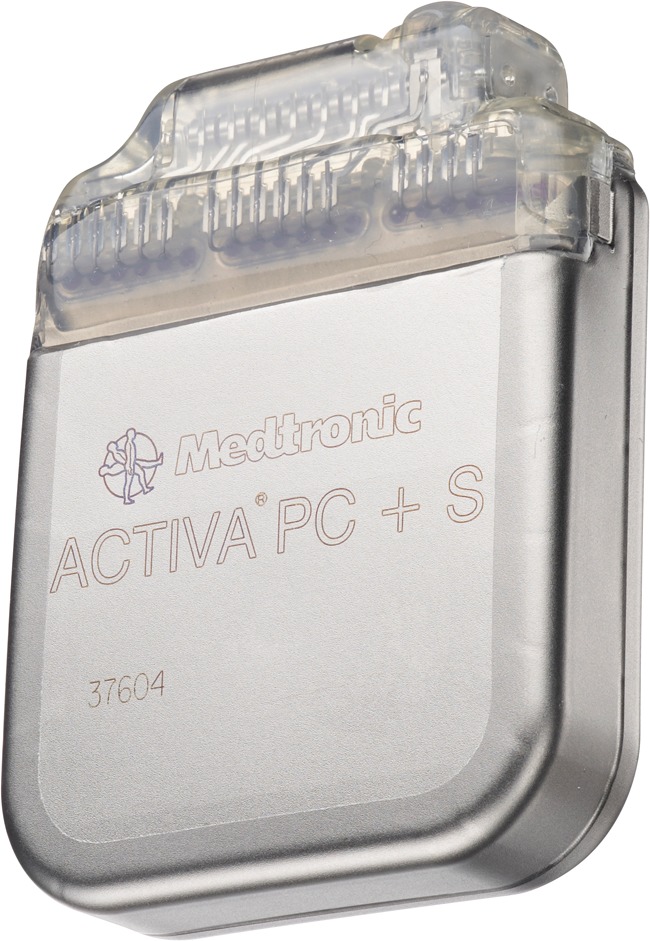
The Activa® PC + S deep brain neurostimulator (Medtronic, Minneapolis, MN, USA). Reprinted with the permission of Medtronic, Inc. © 2013

A fully implantable closed-loop DBS system has recently been developed for investigational use. This system, the Activa PC + S neurostimulator (Medtronic), is based on the Activa PC neurostimulator with additional sensing, stimulation, and detection features (Fig. 3).

The main technical challenge in designing a closed-loop concurrent sensing and stimulation system, in which sensing is performed through the same leads as stimulation, is in separating the stimulation signal (artifact) from the signal of interest. Stanslaski et al. [81, 82] describe a systems-level approach to minimizing the stimulation interference. This approach includes design elements in the sensing component, the stimulation component, and the detection algorithm component.

### Sensing

Three key design elements in the sensing component contribute to the elimination of the stimulation artifact. First, by using differential sensing and electrodes that are symmetric in geometry about the stimulating electrode, a large component of the stimulation artifact can be rejected as a common mode disturbance. Second, a front-end filter can be configured to suppress the high frequency associated with stimulation. For example, a stimulation frequency of 140 Hz can be suppressed with a 100-Hz low-pass filter. Third, a spectral bandpower processor can be used to isolate the signal power in the specific frequency band of interest, thus separating the neural biomarkers from the stimulation harmonics.

### Stimulation

While the design elements in the sensing component are key to suppressing the stimulation artifact in the frequency band of interest, careful selection of the sampling rate and the stimulation frequency are also necessary to separate the stimulation artifact from the signal of interest. Although the stimulation frequency may be higher than the frequency band of interest, the stimulation frequency and its harmonics may be aliased back into lower frequency bands depending on the sampling rate. Therefore, the design includes numerical analysis methods to identify stimulation frequencies that minimize the interference with the band of interest based on the sampling rate.

### Detection Algorithm

Finally, the design includes detection algorithms that further distinguish the information in the signal of interest from the stimulation signal. For example, supplementing the physiological spectral channels with independent information about the stimulation energy may allow a classification algorithm such as a support vector machine to better separate the stimulation signal from the signal of interest.

These design elements, as described by the authors “with modest performance in each individual block but acceptable overall performance” enable measurement of the LFP in the STN during stimulation, thus paving the way for closed-loop DBS.

Proof-of-concept of the concurrent sensing and stimulation design was demonstrated in a large animal (ovine) model with leads implanted in the thalamus and hippocampus [71, 81]. Evoked potentials and LFPs could be recorded during stimulation. Moreover, the evoked potentials were stable for > 1 year, demonstrating the utility of the system as a chronic implant [71].

#### Clinical Experience

The investigational Activa PC + S DBS is currently being used in a clinical trial to study changes in neuronal oscillations during tremor, repetitive movement, and freezing episodes relative to rest in patients with PD, with the first human implants in 2013 [83]. There have been no published reports on experience with the system.

## Discussion

We have reviewed 4 implantable closed-loop neurostimulation systems: positional-adaptive SCS for treatment of pain, responsive cortical stimulation for the treatment of epilepsy, closed-loop VNS for the treatment of epilepsy, and concurrent sensing and stimulation for the treatment of PD. At present, few such systems exist owing to the complexities of designing and implementing such a system. The technical challenges include incorporating physiological sensors that add minimal risk to the patient, and developing algorithms that detect in real-time and require low computational power. The scientific and clinical challenges include determining the physiological markers for specific symptoms, the anatomical target in which to sense and stimulate, the physiological changes to detect, and how to deliver and modulate the stimulation in response to the detected event and, ultimately, the clinical symptoms. However, the clinical experience from these systems support the notion that closed-loop therapy can be more effective than open-loop ones, and therefore underscores the importance of continued effort in developing closed-loop systems and in identifying promising clinical applications. Future applications of closed-loop neurostimulation may include treatment of major depression [84], Tourrette syndrome [85], and other neuropsychiatric disorders. The success of closed-loop SCS for the treatment of pain and closed-loop responsive cortical stimulation for treatment of epilepsy pave the way for development of these other closed-loop neurostimulation systems.

## Electronic supplementary material

Below is the link to the electronic supplementary material.

ESM 1 (PDF 1225 kb)
